# High-flow nasal cannula oxygen therapy versus non-invasive ventilation in healthy respiratory physicians: a non-randomized study

**DOI:** 10.3389/fmed.2024.1506877

**Published:** 2025-01-07

**Authors:** Hong Ye, Dandan Xiang, Xiangyu Zhu, Xiuwei Du, Shengyun Shang, Jing Xu, Yu Li, Yunyun Cheng, Zhongfei Yang

**Affiliations:** ^1^Department of Respiratory and Critical Care Medicine, Qilu Hospital of Shandong University Dezhou Hospital, Dezhou, China; ^2^Department of Ultrasound Medicine, Qilu Hospital of Shandong University Dezhou Hospital, Dezhou, China; ^3^Department of Respiratory and Critical Care Medicine, Qilu Hospital of Shandong University, Jinan, China; ^4^Department of Clinical Laboratory, Qilu Hospital of Shandong University Dezhou Hospital, Dezhou, China

**Keywords:** high-flow nasal oxygen, non-invasive ventilation, comfort, healthy volunteers, flow rate

## Abstract

**Background:**

High-flow nasal cannula (HFNC) and non-invasive ventilation (NIV) are commonly used for respiratory support. This study aims to first establish whether to use HFNC or NIV based on comfort levels, and subsequently evaluate diaphragmatic function under equivalent comfort levels to determine the optimal modality for clinical application.

**Methods:**

A self-controlled, non-randomized study was conducted with 10 healthy respiratory physicians as participants. Each subject was exposed to different HFNC settings, including flow rates of 20, 40, and 60 L/min at both 33 and 37°C. Additionally, participants were assessed under NIV mode. Comfort levels as the primary outcome were evaluated using the Visual Numerical Scale (VNS). Meanwhile, vital signs and diaphragmatic mobility were monitored through an electrocardiograph and ultrasound.

**Results:**

HFNC at a flow rate of 20 L/min provided greater comfort than NIV. However, as the flow rate increased, this comfort benefit decreased. At 40 L/min, comfort levels were similar between HFNC and NIV, while at 60 L/min, HFNC was less comfortable than NIV. Notably, temperature variations between 33 and 37°C had no significant effect on comfort. In addition, under conditions of similar comfort, HFNC demonstrated slightly greater diaphragmatic mobility compared to NIV.

**Conclusion:**

Our study indicated HFNC was the preferred choice for providing respiratory support at low to moderate flow rates in healthy volunteers not requiring respiratory support. By contrast, at higher flow rates, NIV discomfort was lower than HFNC discomfort.

## Introduction

High flow nasal cannula (HFNC) and non-invasive ventilation (NIV) are two oxygenation devices that provide a higher fraction of inspired oxygen (FiO_2_) than traditional oxygen therapies (1–3). HFNC enables delivery of heated and humidified oxygen to the nose at a maximum flow rate of 60 L/min (4–6). The primary mechanisms of HFNC include the generation of positive pressure in the pharynx, the washing out of nasopharyngeal dead space, improving secretion clearance and reducing inspiratory effort (6, 7). NIV assists respiratory support by applying controlled positive pressure to the airways through a non-invasive interface, such as a nasal or oral mask. Several studies have shown that both approaches provide significant clinical benefits for patients with hypoxemic respiratory failure, notably by reducing the need for intubation (3, 8).

Comfort represents a balanced integration of multiple physiological processes, potentially serving as a patient-level outcome *per se* (9, 10). Intolerance to treatment after extubation in COPD patients is associated with the failure of non-invasive respiratory support, highlighting the importance of patient comfort for better clinical outcomes (11). There is ongoing debate regarding the comfort levels of HFNC therapy and NIV. Most studies indicate that HFNC is linked to superior comfort and patient tolerance compared to NIV (11–15), while a few report no significant difference in comfort levels between the two therapies (16, 17). This discrepancy may be attributed to variations in patients’ disease states and individual requirements for flow rates and ventilation modes. Therefore, further research is necessary to explore the differences in comfort levels between HFNC and NIV.

In this project, we enrolled experienced respiratory physicians as healthy volunteers. Our primary objective was to determine whether HFNC or NIV should be selected based on comfort levels, followed by an evaluation of diaphragmatic function under comparable comfort conditions to identify the most suitable modality for clinical application.

## Materials and methods

This was a non-randomized, concurrent, self-controlled study conducted at Qilu Hospital of Shandong University Dezhou Hospital, a tertiary hospital. The trial was approved by our hospital’s institutional review board (No. 2024026). In total, 10 healthy respiratory physicians (5 men, 5 women) aged 24–40 years were enrolled in this study. All participants in this study provided their informed consent. Demographic data, including age, sex, height, weight, and body mass index (BMI), were obtained from all volunteers. We excluded individuals who were pregnant or breastfeeding, had acute upper respiratory infections, pneumonia or rhinitis, suffered from CPAP-related claustrophobia, and had a history of cardiac, pulmonary, hepatic, renal or cerebrovascular diseases. The bi-level non-invasive ventilator R-80S (BMC Medical Co., Ltd., Beijing China), which integrated HFNC and NIV functions, was utilized in the study.

### Study protocol

All physicians rested for 10 min prior to the start of the experiment to attain a physiological steady state. During the study, individuals remained in a 45-degree semi-recumbent position, breathing nasally with their mouth closed (conditioned room air with FiO2 0.21). Using the R-80S device in HFNC mode, the experiment was conducted at two temperature settings (33 and 37°C), with inhaled flow rates gradually adjusted to 20, 40, and 60 L/min. Each combination of parameters was maintained for 5 min, followed by a 5-min washout period between conditions. Following the HFNC phase, participants were transitioned to NIV mode (temperature 33°C, and relative humidity 100%). The initial settings were based on a tidal volume of 6–8 ml/kg, with inspiratory pressures of 8–16 cmH_2_O and positive ernd-expiratory pressures of 4–6 cmH_2_O. The NIV settings were adjusted to achieve a level of relative comfort perceived by the participants, and were then maintained for 5 min. Toward the end of each study phase, we collected data for comfort, heart rate (HR), diastolic blood pressure (DBP), mean arterial pressure (MAP), respiratory rate (RR), peripheral oxygen saturation (SpO2), diaphragm mobility (DM), and thickening fraction (TFdi) through the electrocardiograph monitor (UT6000C, Shenzhen Jingkeway Industrial Co., Ltd., Shenzhen, China).

### Measures

**FIGURE 1:**
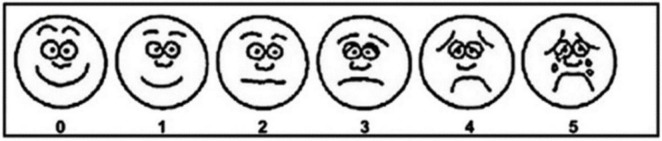
Visual Numerical Scale (VNS) for comfort. 0 = very comfortable; 1 = comfortable; 2 = mild discomfort; 3 = moderate discomfort; 4 = severe discomfort; 5 = unbearable discomfort.

**FIGURE 2:**
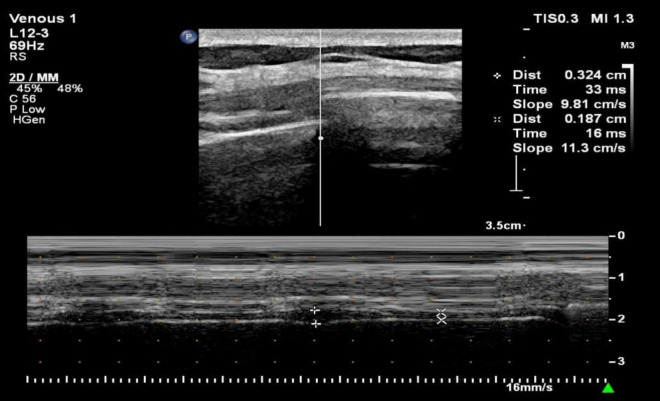
Diaphragm thickness measurement.

**FIGURE 3:**
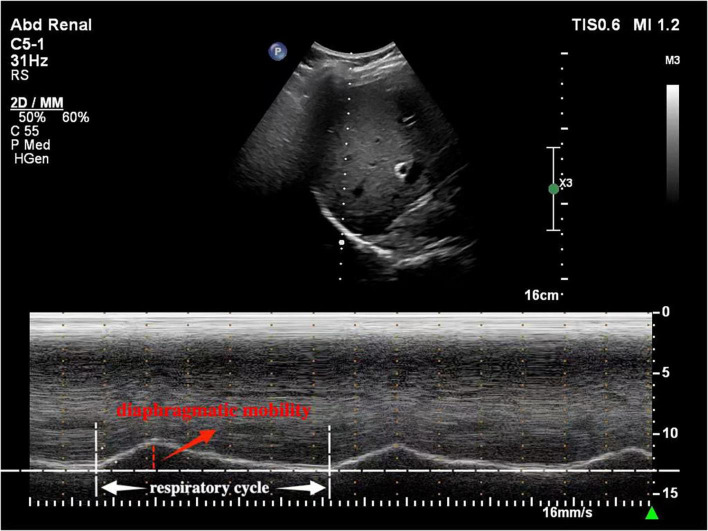
Diaphragm mobility measurement.

The comfort was assessed by Visual Numerical Scale (VNS) ranging from 0 (very comfortable) to 5 (unbearable discomfort) (Figure 1). In fact, the VNS has been widely used to evaluate comfort in clinical studies involving HFNC (2, 18). In addition, diaphragm mobility and thickness were assessed with the Philips EPIQ 7C color Doppler echocardiographer (Philips Healthcare Royal Philips Electronics, Amsterdam, Netherlands). For the evaluation of diaphragm thickness, a 3–12 MHz linear probe was placed between the anterior axillary line and the mid-axillary line to obtain a sagittal image of the intercostal space between the 8th to 9th ribs. Diaphragm thickness in individuals was measured twice, at the end of inspiration (Tdi) and at the end of expiration (Tde). The diaphragm thickening fraction (TFdi) was calculated using the formula: TFdi = (Tdi–Tde)/Tde × 100% (19) (Figure 2). The measurement of diaphragm mobility was performed using a 1–5 MHz curved probe. The transducer was positioned over the right subcostal area, with the ultrasound beam angled toward the cranio-caudal axis to identify the left portal vein branch as a reference point (20). The diaphragm mobility was represented by the displacement from the lowest point at the end of inspiration to the highest point during maximal inspiration (Figure 3). All measurements were carried out three times with the mean value applied.

### Statistical analysis

The enrollment of 10 healthy physicians was planned (study power = 0.8, α = 0.05) based on a clinically meaningful difference of 2.0 ± 1.5 points in comfort among different groups (21). The data were analyzed using SPSS 27.0 (IBM Corp., Armonk, NY, USA) packaged software. Data following a normal distribution, as confirmed by Kolmogorov-Smirnoff, were presented as mean ± standard error. Otherwise, they were expressed as median and interquartile range. The Wilcoxon signed-rank test was employed to compare the comfort levels between different groups, and the differences in vital signs, diaphragm mobility, and diaphragm thickening ratio were analyzed using paired t-tests. The value of *p* < 0.05 was considered to be statistically significant in all analyses.

## Results

### Participants characteristics

In total, 10 healthy physicians (5 men and 5 women) were included in this study. Their mean age was 33.60 ± 5.72 years, with a height of 165.50 ± 6.47 cm, weight of 70.70 ± 8.13 kg and BMI of 25.72 ± 2.31 kg/m^2^ (Table 1).

**TABLE 1:** Baseline characteristics of the study population.

	Total	Male	Female
Age (years)	33.60 ± 5.72	32.20 ± 5.22	35.00 ± 6.44
Height (cm)	165.50 ± 6.47	171.00 ± 2.24	160.00 ± 3.67
Body weight (kg)	70.70 ± 8.13	74.40 ± 4.72	67.00 ± 9.59
BMI (kg/m^2^)	25.72 ± 2.31	25.36 ± 1.31	26.08 ± 3.15
Total	10	5	5

Values are presented as mean ± standard error.

### Differences in comfort between HFNC and NIV devices

**FIGURE 4:**
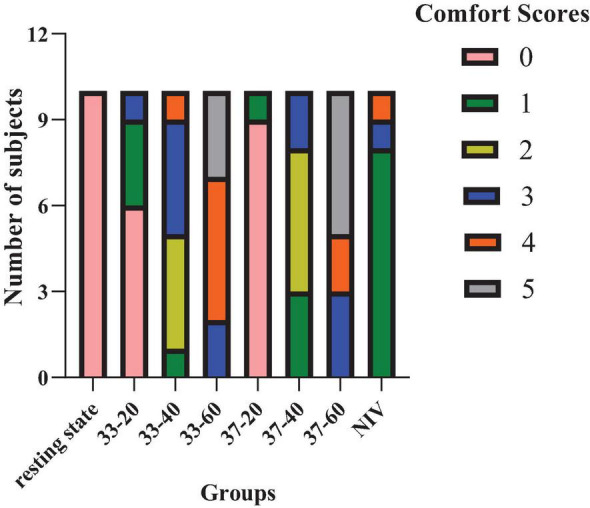
The effect of HFNC and NIV devices on comfort. 33-20 represents HFNC mode with a temperature of 33°C and a flow rate of 20 L/min. Other combinations follow the same format.

There was no significant difference in comfort between resting state and HFNC modes at 33 and 37°C with 20 L/min, whereas NIV was associated with significant higher discomfort (*p* < 0.01). The comfort of the participants was significantly higher at the lower flow rates in comparison with the higher flow rates (*p* < 0.01). Specifically, HFNC at 20 L/min provided superior comfort compared to NIV. When the rate flow reached 40 L/min, comfort levels were similar between the two devices, but at flow rates of 60 L/min, the comfort level of HFNC was lower than that of NIV (Figure 4 and Table 2). However, comfort was not affected by temperature. Overall, HFNC demonstrated better comfort at lower flow rates, but as the flow rate increased, its comfort level declined, eventually becoming less favorable than NIV at higher flow rates.

**TABLE 2:** Comfort assessments.

Groups	Resting state	33-20	33-40	33-60	37-20	37-40	37-60	NIV
Resting state	_	−1.890	−2.840	−2.850	−1.000	−2.850	−2.850	−2.970
		0.059	**0.005**	**0.004**	0.317	**0.004**	**0.004**	**0.003**
33-20	−1.890	_	−2.836	−2.840	−1.633	−2.516	−2.848	−2.714
	0.059		**0.005**	**0.005**	0.102	**0.012**	**0.004**	**0.007**
33-40	−2.840	−2.836	_	−2.889	−2.848	−1.613	−2.588	−2.226
	**0.005**	**0.005**		**0.004**	**0.004**	**0.107**	**0.01**	**0.026**
33-60	−2.850	−2.840	−2.889	_	−2.873	−2.842	−0.447	−2.827
	**0.004**	**0.005**	**0.004**		**0.004**	**0.004**	0.655	**0.005**
37-20	−1.000	−1.633	−2.848	−2.873	_	−2.877	−2.836	−2.807
	0.317	0.102	**0.004**	**0.004**		**0.005**	**0.005**	**0.005**
37-40	−2.850	−2.516	−1.613	−2.842	−2.877	_	−2.831	−1.081
	**0.004**	**0.012**	0.107	**0.004**	**0.005**		**0.005**	0.279
37-60	−2.850	−2.848	−2.588	−0.447	−2.836	−2.831	_	−2.831
	**0.004**	**0.004**	**0.01**	0.655	**0.005**	**0.005**		**0.005**
NIV	−2.970	−2.714	−2.226	−2.827	−2.807	−1.081	−2.831	_
	**0.003**	**0.007**	**0.026**	**0.005**	**0.005**	0.279	**0.005**	

33-20 represents HFNC mode with a temperature of 33°C and a flow rate of 20 L/min. Other combinations follow the same format. The upper values in each cell represent Z-scores, while the lower values correspond to *p*-values. *p*-values that are statistically significant (*p* < 0.05) are highlighted in bold.

### The effects of HFNC and NIV devices on vital signs and diaphragm mobility and thickening ratio

A significant reduction in RR was observed at 40 L/min compared to the resting state at 33°C. Similarly, at 37°C, both HR and RR significantly decreased at 20 and 40 L/min in contrast to the resting state, with RR at 40 L/min also showing a notable difference from NIV. Additionally, NIV demonstrated a notable impact on HR and SpO2 compared with the resting state (Table 3).

**TABLE 3:** Comparison of vital signs and diaphragm parameters among different groups.

Variable		33°C			37°C		Resting state	NIV
	**20L/min**	**40L/min**	**60L/min**	**20L/min**	**40L/min**	**60L/min**		
HR	72.30 ± 3.80	72.00 ± 7.30	73.50 ± 6.90	71.4 ± 34.50^a^	70.60 ± 8.80^a^	72.50 ± 3.70	76.00 ± 8.80	71.00 ± 5.50^a^
RR	14.90 ± 5.44	12.95 ± 3.95 ^a^	13.30 ± 4.87	12.90 ± 2.51^a^	11.80 ± 2.78^ab^	12.60 ± 3.41^a^	16.50 ± 3.27	15.40 ± 1.96
MAP	89.70 ± 11.06	88.90 ± 13.65	92.00 ± 12.8	86.10 ± 13.44	87.00 ± 13.27	91.90 ± 16.01	89.70 ± 11.06	87.4 ± 13.27
SpO_2_	95.50 ± 1.18	98.80 ± 1.23	99.00 ± 1.25	98.80 ± 0.79	99.10 ± 0.99	99.20 ± 0.92	98.50 ± 1.18	99.60 ± 0.69^a^
DM	1.49 ± 0.48	1.69 ± 0.65	1.50 ± 0.54	1.36 ± 0.62	1.63 ± 0.52	1.45 ± 0.66	1.59 ± 0.62	1.54 ± 0.58
TFdi	0.54 ± 0.12	0.58 ± 0.24	0.55 ± 0.21	0.52 ± 0.23	0.57 ± 0.24	0.60 ± 0.29	0.52 ± 0.21	0.52 ± 0.17

Values are presented as mean ± standard error, “a” represents *p* < 0.05 compared to the resting state, and “b” indicates *p* < 0.05 compared to NIV. HR, heart ratio; RR, respiratory rate; MAP, mean arterial pressure; SpO2, oxygen saturation; DM, diaphragm mobility; TFdi, diaphragm thickening fraction.

Diaphragm mobility increased at 33°C and 40 L/min, as well as at 37°C and 40 L/min compared to NIV, but without statistical difference. There were also no significant differences in diaphragm thickening ratio across all parameters (Table 3).

## Discussion

In the present investigation, we evaluated the comfort levels between high flow nasal cannula (HFNC) and non-invasive ventilation (NIV), as well as their impacts on vital signs and diaphragmatic function in healthy respiratory physicians. To our knowledge, this is the first study to directly compare these two respiratory support devices in a population of respiratory physicians serving as healthy subjects.

Our results revealed that HFNC provided greater comfort than NIV at 20 L/min, supporting the advantage of HFNC as a preferred respiratory support tool at lower flow rates. However, as the flow rate increased to 40 L/min and 60 L/min, comfort levels declined, with HFNC being less comfortable than NIV, particularly at 60 L/min. This finding is consistent with previous reports (22–25). The increased flow rate resulted in alveolar overdistention in non-dependent lung regions and prolonged exhalation, which might be the reason for the nasal discomfort. In addition, in our study, the comfort levels of HFNC was not affected by temperature. It’s noteworthy that Mauri et al. also assessed comfort during HFNC at increasing flow and temperature in acute hypoxemic respiratory failure (AHRF) patients (18). They found that at equal flow rates, lower temperatures provided greater comfort, and higher flow rates did not reduce comfort. In fact, higher flow improved comfort in more severely hypoxemic patients. The difference observed between our study and that of Mauri et al. is likely due to variations in participant health status. Patients with more severe hypoxemia require higher FiO2, which leads to more effective correction of hypoxemia and improved lung mechanics (26–28), thereby leading to increased comfort. In contrast, healthy individuals with normal respiratory function can tolerate a maximum flow rate of 30 L/min (29). To this end, our findings might suggest that HFNC is more suitable for patients with lower flow rate requirements, whereas NIV could be a better option for those requiring higher flow rates.

In addition, in our study, HFNC at 40 L/min slightly increased diaphragmatic mobility compared to NIV, likely due to reduced respiratory rate and improved breathing efficiency. At 60 L/min, the slight decrease in diaphragmatic mobility was likely linked to prolonged exhalation or involuntary adjustments in breathing patterns caused by reduced comfort. Further studies are needed to confirm these findings in clinical patient populations.

A major strength of this study is that it employs a rigorous self-controlled design, which effectively minimizes inter-subject variability and enhances the reliability of the results. Besides, by using respiratory physicians as the participant group, this study ensures a high degree of compliance and procedural familiarity, thereby minimizing potential confounding factors such as anxiety or unfamiliarity with the devices, which are commonly observed in patient-based studies.

However, this study has several limitations. First, the small sample size, along with the short duration of device use, may affect the statistical validity and restrict the applicability of the findings. Furthermore, the selection of healthy physicians as participants could have introduced potential biases in subjective comfort evaluations due to their professional expertise and practical experience with similar medical interventions. Additionally, the use of healthy participants may not adequately represent the physiological and comfort responses observed in patients with respiratory conditions. Finally, the 5-min periods of ventilation at each HFNC flow rate may cause cumulative fatigue or adaptation, potentially affecting comfort evaluations and diaphragmatic mobility measurements. Future studies with larger, more diverse patient populations and randomized designs are needed to validate these findings and better understand the clinical implications of HFNC and NIV in different disease contexts.

## Conclusion

This study was conducted as a non-randomized trial to compare the effects of HFNC and NIV on comfort and diaphragmatic function. In healthy volunteers, HFNC provided greater comfort compared to NIV at lower flow rates (20 L/min). However, at higher flow rates (60 L/min), NIV discomfort was lower than HFNC discomfort.

## Data Availability

The original contributions presented in this study are included in this article/supplementary material, further inquiries can be directed to the corresponding authors.
